# Fabrication and Cytotoxicity of Gemcitabine-Functionalized Magnetite Nanoparticles

**DOI:** 10.3390/molecules22071080

**Published:** 2017-06-28

**Authors:** Roxana Cristina Popescu, Ecaterina Andronescu, Bogdan Ștefan Vasile, Roxana Truşcă, Adina Boldeiu, Laurențiu Mogoantă, George Dan Mogoșanu, Mihaela Temelie, Mihai Radu, Alexandru Mihai Grumezescu, Diana Savu

**Affiliations:** 1 Department of Life and Environmental Physics, “Horia Hulubei” National Institute for Physics and Nuclear Engineering, 30 Reactorului Street, Măgurele 077125, Romania; roxana.popescu@nipne.ro (R.C.P.); mihaela.temelie@nipne.ro (M.T.); mradu@nipne.ro (M.R.); 2 Department of Science and Engineering of Oxide Materials and Nanomaterials, Faculty of Applied Chemistry and Materials Science, University Politehnica of Bucharest, 1–7 Polizu Street, Bucharest 011061, Romania; ecaterina.andronescu@upb.ro (E.A.); bogdan.vasile@upb.ro (B.Ș.V.); truscaroxana@yahoo.com (R.T.); 3 Laboratory of Nanobiotechnology, National Institute for Research and Development in Microtechnologies, 12A Erou Iancu Nicolae Street, Bucharest 077190, Romania; adina.bragaru@imt.ro; 4 Research Center for Microscopic Morphology and Immunology, University of Medicine and Pharmacy of Craiova, 2 Petru Rareș Street, Craiova 200349, Romania; editor@rjme.ro; 5 Department of Pharmacognosy and Phytotherapy, Faculty of Pharmacy, University of Medicine and Pharmacy of Craiova, 2 Petru Rareș Street, Craiova 200349, Romania; mogosanu2006@yahoo.com

**Keywords:** magnetite nanoparticles, Gemcitabine, cancer therapy, in vitro cytotoxicity, biodistribution

## Abstract

Nanotechnology has been successfully used for the fabrication of targeted anti-cancer drug carriers. This study aimed to obtain Fe_3_O_4_ nanoparticles functionalized with Gemcitabine to improve the cytotoxic effects of the chemotherapeutic substance on cancer cells. The (un) functionalized magnetite nanoparticles were synthesized using a modified co-precipitation method. The nanoconjugate characterization was performed by XRD, SEM, SAED and HRTEM; the functionalizing of magnetite with anti-tumor substances has been highlighted through TGA. The interaction with biologic media has been studied by means of stability and agglomeration tendency (using DLS and Zeta Potential); also, the release kinetics of the drug in culture media was evaluated. Cytotoxicity of free-Gemcitabine and the obtained nanoconjugate were evaluated on human BT 474 breast ductal carcinoma, HepG2 hepatocellular carcinoma and MG 63 osteosarcoma cells by MTS. In parallel, cellular morphology of these cells were examined through fluorescence microscopy and SEM. The localization of the nanoparticles related to the cells was studied using SEM, EDX and TEM. Hemolysis assay showed no damage of erythrocytes. Additionally, an in vivo biodistribution study was made for tracking where Fe_3_O_4_@Gemcitabine traveled in the body of mice. Our results showed that the transport of the drug improves the cytotoxic effects in comparison with the one produced by free Gemcitabine for the BT474 and HepG2 cells. The in vivo biodistribution test proved nanoparticle accumulation in the vital organs, with the exception of spleen, where black-brown deposits have been found. These results indicate that our Gemcitabine-functionalized nanoparticles are a promising targeted system for applications in cancer therapy.

## 1. Introduction

Despite its adverse effects (systemic toxicity, lack of targeting, treatment resistance), chemotherapy is the most used method in cancer treatment. Many research approaches are done to improve the efficiency of this type of treatment concomitantly decreasing its side-effects.

Gemcitabine (GEM) is a cytostatic drug used for the treatment of several types of cancer, such as breast cancer, hepatocarcinoma, pancreatic cancer, ovarian cancer, lungs cancer and also in the treatment of doxorubicin-resistant osteosarcoma. GEM is an analogue nucleoside which acts as an anti-metabolite with chemotherapeutic effect, inhibiting the DNA replication, by blocking the replication process and the repairing mechanisms of the tumor cell [1]. GEM replaces the cytidine residue from the DNA replication block, during the replication process. Due to its rapid enzymatic degradation after intravenous injection, GEM has a short blood half-life. This is one of the main drawbacks of using GEM in chemotherapy [2]. For this reason, it is necessary to administer high drug doses to reach the required therapeutic response, thus increasing the side-effects.

To increase the local concentration of GEM at the tumor site and, thus, the cytotoxic effects, one proposed solution consists in using nanoparticles as passive or active carriers for chemotherapeutic substances [3,4]. Passive targeting refers to the enhanced permeability and retention effect (EPR) at the tumor site. This is caused by the fact that the tumor tissues are more perfused, blood vessel walls having many abnormalities, including high level of fenestration, allowing the nanoparticles to pass through this barrier. On the other hand, active targeting can be done either by magnetic guiding, either by applying different functionalizing agents that specifically bind to receptors on the surface of the targeted cancer cells.

Today, there are few Food and Drug Administration−approved nanoconjugates for clinical use, however, there is still a tremendous need to find more effective treatment options, which will overcome the shortcomings of the existing ones. One attempt to overcome tumor resistance to radiotherapy and/or chemotherapy was by using appropriate carrier systems to increase drug delivery at tumor site. Nowadays, the use of magnetic nanoparticles as drug transporters has been an area of significant interest [5]. Besides its biocompatibility, one reason for using magnetite Fe_3_O_4_ nanoparticles in this type of delivery system, is given by the possibility of applying any of the previously mentioned targeting approaches: passive by EPR [6], and active by magnetic targeting [7,8,9,10] or specific functionalizing [11,12,13,14]. Fe_3_O_4_ nanoparticles have been already used as carriers for GEM in some in vitro approaches aiming to improve the drug release time [15], and efficiency [16,17].

In the present study, a novel nanoconjugate based on magnetite nanoparticles functionalized with GEM was developed and characterized to be used as anticancer agent. The main purpose for attaching Gemcitabine to the surface of Fe_3_O_4_ nanoparticles was to obtain a system which improves the cytotoxic effects of the chemotherapeutic substance for cancer cells. The structural and compositional characterization of this nanoconjugate was accomplished through methods such as X-ray diffraction (XRD), Scanning electron microscopy (SEM), Selected Area Electron Diffraction (SAED) and High Resolution Transmission Electron Microscopy (HRTEM) analysis, while the functionalizing of magnetite with anti-tumor substances has been highlighted through the thermogravimetric analysis (TGA). The biologic evaluation of this novel nanoconjugate was performed by in vitro testing of the cytotoxic potential against three types of cancer cells, which differ depending on the organ of origin. Hemolysis assay showed no damage of erythrocytes. Additionally, in vivo biodistribution studies were done on mice models.

## 2. Results and Discussion

### 2.1. Nanoparticle Synthesis

The most encountered method to obtain magnetite nanoparticles is the co-precipitation, due to its reproducibility and simplicity [4]. Here, a modified Massart’s method [18] was employed in order to obtain Gemcitabine functionalized magnetite nanoparticles (Fe_3_O_4_@GEM) with application in cancer treatment.

Traditionally, the method starts from two precursors of iron with molar ratio Fe^3+^: Fe^2+^ of 2:1, however a smaller ratio can be used to compensate the oxidation of Fe^2+^ [19,20]. Jiang et al. [20] has evaluated the influence of Fe^3+^: Fe^2+^ molar ratio on the properties of iron oxide nanoparticles obtained using a derived co-precipitation method. When the ratio is below 1.75:1, the nanoparticles’ phase composition is pure magnetite, while the best magnetic properties are given by the samples obtained at around 1.5:1 molar ratio between the precursors. Here, a ratio of 1.65:1 was employed.

An ammonia solution was also used to set the pH value at around 11, because it is a weak base that partially ionizes in the aqueous precipitation media and also enriches the concentration of N_2_, which helps preventing the oxidation.

It is commonly encountered that an organic phase is introduced in the precipitation medium, in order to form small micelles as nucleation space for the nanoparticles, which is known as microemulsion method [21]. These control the dimension of the final particles and determine the forming of a direct coating on the surface of the material. In order to avoid a subsequent step of functionalizing, we introduced the chemotherapeutic substance in the precipitation medium to facilitate the direct functionalizing with the organic molecule, during the growth process of the nanoparticles.

### 2.2. Structural Characterization

#### 2.2.1. X-ray Diffraction Analysis

**Figure 1:**
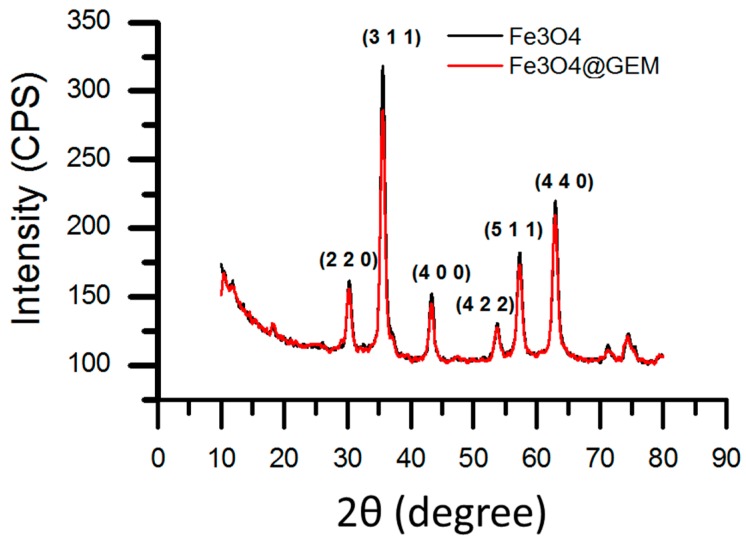
XRD pattern of Fe_3_O_4_ and Fe_3_O_4_@GEM; the characteristic diffraction interferences of magnetite phase are presented in different regions of 2θ, corresponding to the indices: 18.31° (111), 30.33° (220), 35.51° (311), 43.16° (440), 53.61° (422), 57.17° (511), 62.82° (440), 74.24° (533).

The crystallinity degree characterization and the identification of the resulted samples were done using X-ray diffraction XRD analysis (Figure 1); the diffraction interferences were indexed using JCPDS file no. 19-0629 as corresponding to magnetite, the results being in concordance with previously reported data [22]. Eight magnetite characteristic peaks were presented in the obtained spectrum, in different regions of 2θ, corresponding to the following indices: 18.31° (111), 30.33° (220), 35.51° (311), 43.16° (440), 53.61° (422), 57.17° (511), 62.82° (440), 74.24° (533). The presence of the organic chemotherapeutic substance did not affect the crystallinity of the nanoparticles, nor induce other significant changes in the phase composition of the samples.

#### 2.2.2. Thermogravimetric Analysis

**Figure 2:**
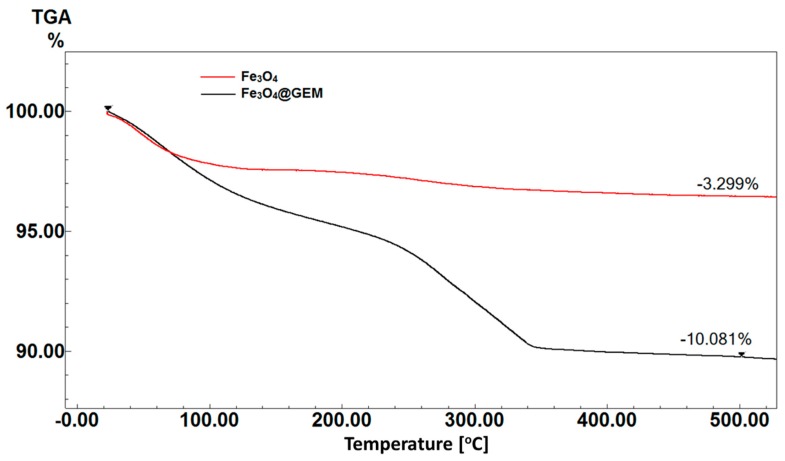
TGA curves for Fe_3_O_4_ and Fe_3_O_4_@GEM; the weight reduction evidenced for Fe_3_O_4_ sample was 3.3%, while for Fe_3_O_4_@GEM was 10.1%.

The thermogravimetric analysis was used to measure the quantity of the organic gemcitabine phase interacting with the inorganic Fe_3_O_4_ nanoparticles. Thus, by applying several heating cycles for both Fe_3_O_4_ and Fe_3_O_4_@GEM, the existing quantity of GEM in the functionalized sample was calculated from the mass differences (Figure 2). The first weight loss threshold identified is below the temperature of approximately 250 °C and is attributed to the evaporation of the absorbed water on the nanoparticles’ surface (water thermos-desorption [23]). The degradation of the organic chemotherapeutic takes place in two steps, between 250–350 °C and 350–500 °C. This supplementary threshold may be due to the degradation of hydroxyl groups on the surface of the nanoparticles [23]. Similarly, Villa et al. [24] have reported a two-step degradation of aminopropyltrietoxysilane (APTES) linked to the surface of Fe_3_O_4_ nanoparticles. Here, for Fe_3_O_4_, a weight reduction of 3.3% was obtained and for Fe_3_O_4_@GEM 10.1%, from which 6.8% is due to the degradation of GEM.

#### 2.2.3. Scanning Electron Microscopy

**Figure 3:**
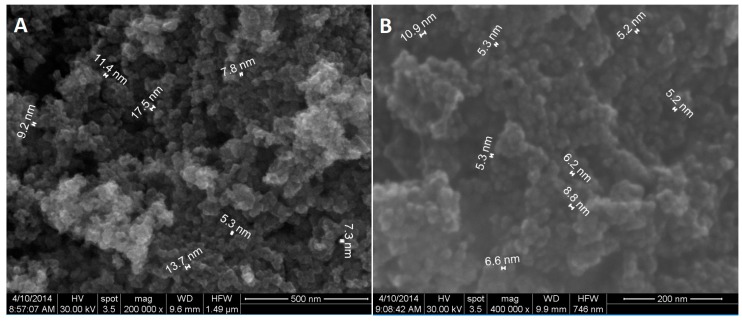
SEM images of Fe_3_O_4_ nanoparticles (**A**) and Fe_3_O_4_@GEM (**B**).

In order to obtain information about the nanoparticles’ morphology (shape and dimension) scanning electron microscopy SEM was used at first (Figure 3). The aspect of the powders is uniform, consisting in aggregates of spheroid nanoparticles with dimensions between 4 and 20 nm.

#### 2.2.4. Transmission Electron Microscopy

**Figure 4:**
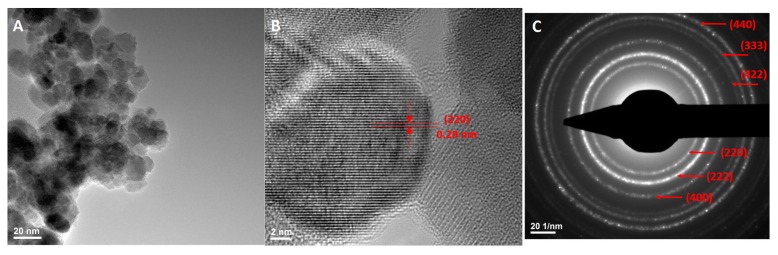
(**A**) TEM images, (**B**) HRTEM images and (**C**) SAED spectrums for Fe_3_O_4_ nanoparticles.

**Figure 5:**
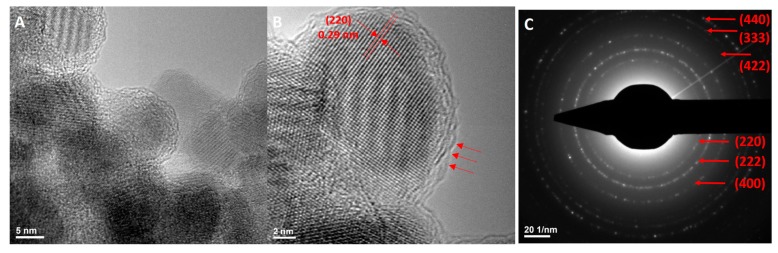
(**A**) TEM images, (**B**) HRTEM images and (**C**) SAED spectrums for Fe_3_O_4_@GEM nanoparticles.

Transmission electron microscopy TEM (Figure 4 and Figure 5) gave more information about the morphology and crystallinity of the nanoparticles, both functionalized and unfunctionalized. From high resolution TEM (HR-TEM, Figure 4 and Figure 5B) a high degree of crystallinity of both samples was observed, in case of Fe_3_O_4_@GEM nanoparticles, GEM individually covering each nanoparticle forming a continuous amorphous layer on the surface of Fe_3_O_4_, resulting core-shell structures. Figure 4B and Figure 5B emphasize the (220) crystalline plan of 0.29 nm, characteristic of a magnetite phase [25].

By means of structural characteristics, the high level of crystallinity deduced from HR-TEM analysis was also confirmed by Selected Area Electron Diffraction SAED patterns (Figure 4 and Figure 5C), from which the samples were identified as being face-centered magnetite with spinel structure, according to previously published data [26,27].

### 2.3. Interaction with Biologic Media

#### 2.3.1. Dynamic Light Scattering and Zeta Potential

Recent studies reveal various behavior of nanoparticles, in terms of agglomeration and even aggregation, when they are introduced in cell culture media, enriched with different amounts of proteins, amino acids, electrolytes, etc. [28]. As a result, the in vitro and in vivo behavior of the nanoparticles is altered, an important issue to be solved and understood being the nanoparticles’ colloidal stability, following their interaction with different biological media.

In this study the behavior of GEM functionalized Fe_3_O_4_ nanoparticles in two cell culture media, DMEM and MEM, was evaluated by investigating the nanoparticles’ stability and hydrodynamic diameters. The interval of dilutions was chosen to be in concordance with the concentrations employed in the MTS viability assessment experiments (from 0.25 to 0.025% of nanoparticles). First of all, the high colloidal stability of the stock solution (1% nanoparticles in distilled water) must be highlighted, given by the excellent zeta potential value, −32.69 mV; the mean hydrodynamic diameter of Fe_3_O_4_@GEM stock solution was 176.9 (±15). The hydrodynamic diameters of the Fe_3_O_4_@GEM in complete DMEM are comprised in the interval of 218–280 nm and the Zeta Potential between −12 and −23 mV for the investigated range of concentration.

The Fe_3_O_4_@GEM nanoparticles suspended in complete MEM showed values of the Zeta Potential above 20 mV, showing an increased stability compared to the case of DMEM suspensions. In this case, the hydrodynamic diameter was comprised between 238 nm and 270 nm.

Another important aspect is related to the dispersion state measured by means of polydispersity index. Here, in all cases, for both MEM and DMEM, the polydispersity indexes were lower than 0.3, which is an indicative of monodisperse systems [29]. The negative charge of the nanoparticles is in agreement with previously reported data [30], however the suspensions in MEM culture media are more stable than DMEM based suspensions. The increase of the hydrodynamic diameter for both types of dispersions (compared to stock solutions) is given by the adsorption of the serum proteins on the nanoparticles’ surface, which is more abundant in case of DMEM, but also due to the high ion content of the culture media [28]. Being enriched with more proteins and salts with high ionic strength, there might be a certain suppression of the electrostatic double layer in the case of DMEM, which decreases the electrostatic repulsion forces, thus the DMEM based dispersions show instability and subsequently precipitate [31]. In this regards, Sabuncu et al. [32] have evaluated the interaction of gold nanoparticles in DMEM culture media supplemented with 10% FBS, resulting in high aggregates with mean diameters of 400 nm and low stability (zeta potential of about 15 mV).

#### 2.3.2. Release Kinetics

**Figure 6:**
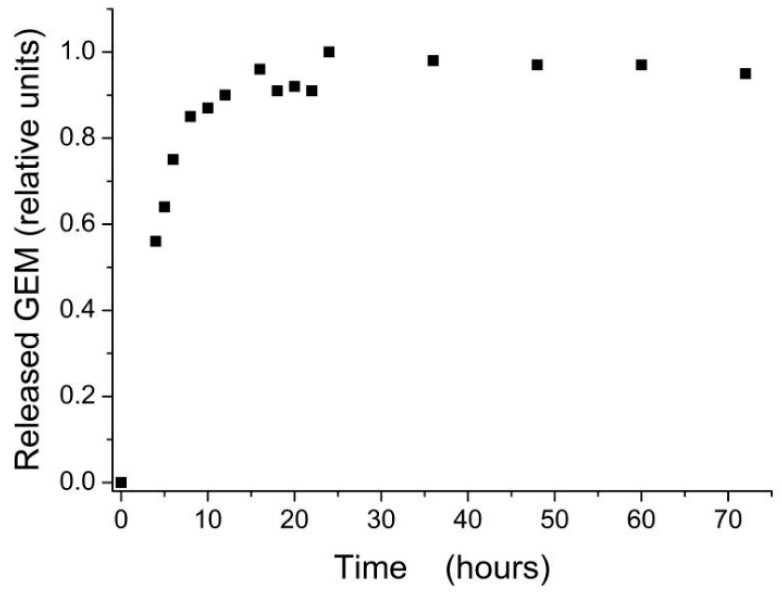
Release kinetics of Gemcitabine drug from Fe_3_O_4_@GEM nanoparticles in complete DMEM culture media, at 37 ± 2 °C, 5 ± 1% CO_2_, more than 90% humidity.

An essential issue in using the drug conjugated nanoparticles for cell cultures treatments is the kinetics of drug release in culture medium. The GEM functionalized magnetite nanoparticles prove to release the drug in surrounding medium during the first 24 h (Figure 6). This slow release rate potentially assures a longer presence of drug in the blood stream, at in vivo level, this being one of the advantages given by the conjugation of drugs to nanoparticles surfaces in drug delivery applications.

### 2.4. Biologic Evaluation

**Figure 7:**
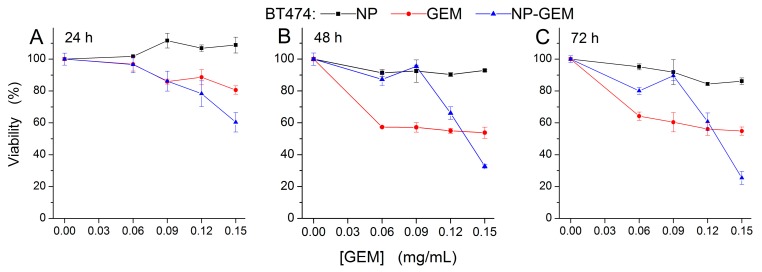
Cell viability of BT-474 cells, incubated with free GEM and Fe_3_O_4_@GEM (equivalent GEM concentrations), determined at (**A**) 24 h; (**B**) 48 h and (**C**) 72 h after the treatment. Data are expressed as percentage of the untreated control. Two-ways ANOVA statistical analysis revealed a significant difference between the GEM and NP-GEM experimental conditions (F(1) = 11, *p* < 0.01 at 24 h; F(1) = 118, *p* < 0.001 at 48 h; F(1) = 9, *p* < 0.01 at 72 h;). The GEM concentration variation along the investigated range produced significant reduction of the viability (F(4) = 33, *p* < 0.001 at 24 h; F(4) = 305, *p* < 0.001 at 48 h; F(4) = 219, *p* < 0.001 at 72 h) irrespective of nanoparticles presence.

**Figure 8:**
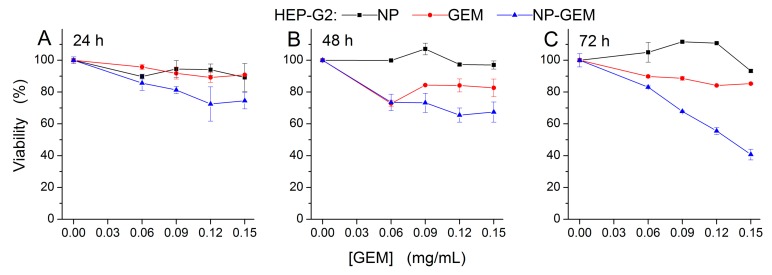
Cell viability of Hep-G2 cells, incubated with free GEM and Fe_3_O_4_@GEM (equivalent GEM concentrations), determined at (**A**) 24 h; (**B**) 48 h and (**C**) 72 h after the treatment. Data are expressed as the percentage of the untreated control. Each data point represents the mean ± SD of three experiments. Two-ways ANOVA statistical analysis revealed a significant difference between the GEM and NP-GEM experimental conditions (F(1) = 42, *p* < 0.001 at 24 h; F(1) = 72, *p* < 0.001 at 48 h; F(1) = 566, *p* < 0.01 at 72 h;). The GEM concentration variation along the investigated range produced significant reduction of the viability (F(4) = 17, *p* < 0.001 at 24 h; F(4) = 9, *p* < 0.001 at 48 h; F(4) = 234, *p* < 0.001 at 72 h) irrespective of nanoparticles presence.

**Figure 9:**
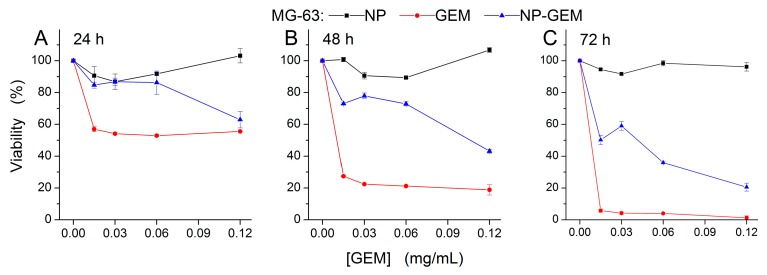
Cell viability of MG-63 cells incubated with free GEM and Fe_3_O_4_@GEM (equivalent GEM concentration), determined at: (**A**) 24 h; (**B**) 48 h and (**C**) 72 h, after the treatment. Data are expressed as the percentage of the untreated control. Each data point represents the mean ± SD of three experiments. Two-ways ANOVA statistical analysis revealed a significant difference between the GEM and NP-GEM experimental conditions (F(1) = 327, *p* < 0.001 at 24 h; F(1) = 5429, *p* < 0.001 at 48 h; F(1) = 2722, *p* < 0.01 at 72 h;). The GEM concentration variation along the investigated range produced significant reduction of the viability (F(4) = 150, *p* < 0.001 at 24 h; F(4) = 2341, *p* < 0.001 at 48 h; F(4) = 3033, *p* < 0.001 at 72 h) irrespective of nanoparticles presence.

The cytotoxicity of GEM functionalized magnetite nanoparticles (NP-GEM) has been evaluated on three frequently used human tumor models: BT 474 human breast ductal carcinoma (Figure 7), HepG2 human hepatocellular carcinoma (Figure 8) and MG 63 human osteosarcoma cells lines (Figure 9). An in vitro assay based on the tetrazolium-salt addressing cells viability was chosen, being currently used in similar studies [22,33]. The cytotoxic effect was characterized by comparison with two other experimental conditions of cells: magnetite nanoparticles (NP) and treatment with Gemcitabine alone (GEM) for three periods of cell treatment (24 h, 48 h and 72 h). The GEM concentration in NP-GEM treatments was computed using the TGA measurements as described in the Materials and Methods).

For all three cell lines, the bare Fe_3_O_4_ did no significant viability changes for all the investigated periods of exposure (Figure 7, Figure 8 and Figure 9, black square graphs). Indeed, the magnetite nanoparticles proved to be biocompatible (viability was not less than 80%, according to ISO 10993-12:2001(E)). These data confirmed previous results which shown the biocompatibility property of the bare magnetite [34,35,36].

For these reason, in the statistical evaluation a two-ways ANOVA was used with presence/absence of nanoparticles being the first factor and the GEM concentration as the second one.

As a common behaviour, in all three celll lines, the GEM and GEM funtionalized nanoparticles produce, as expected, stronger effects at longer periods of treatment. Moreover, the two-ways ANOVA analysis reveled a strong interaction between the analysed factors, the singnificance of this interaction being enhanced for longer periods of treatment (F(4) > 5, *p* < 0.01), for all the investigated conditions. This highly significant interaction between the investigated factors proves the fact that GEM conjugated on magnetite nanoparticles has a different way of interaction with the cells, comparing with free GEM, probably due to the release kinetics described above (Section 2.3.2). However distinct differences among cells’ responses have been observed.

In case of breast cancer cell line BT 474 (Figure 7), results showed that free GEM induced a slight decrease of cell viability (viability of GEM treated cells being greater than 80%, compared to control), starting at 24 h after the treatment, for the highest concentration used (0.15 mg/mL). Then, at 48 and 72 h, it became cytotoxic (cell viability around 60%, compared to control), being independent to the dose of chemotherapeutic. Moreover, the longer exposure (72 h) did not increase the cytotoxic effect of GEM, compared to 48 h exposure condition. The GEM nanostructured conjugate had a cytotoxic effect (viability decreased at 60%, compared to control), starting at 24 h after the treatment, for the highest equivalent concentration used (0.15 mg/mL); then, the anticancer effect became more pronounced with time, at 48 h and 72 h after the treatment, the viability dropping lower than 80% for 0.12 mg/mL and lower than 40% for 0.15 mg/mL. The drug activity, either free or conjugated to nanoparticles, was found to be dose and time dependent. A potentiating effect for the nanoconjugate, compared to free GEM, was observed for the highest concentration (0.15 mg/mL Gemcitabine equivalent concentration) at all time intervals used (Figure 7).

Consistent with our findings, Parsian et al. [37] reported that loading of GEM on chitosan magnetic nanoparticles increased the drug cytotoxic effect on SKBR and MCF-7 breast cancer cells. At 72 h after the treatment, as the viability decreased way under 40%, a highly cytotoxic effect of the systems was shown.

On the other hand, in the range of lower concentrations (under 0.12 mg/mL) the nanoparticles inhibit the effect of GEM with respect to GEM alone. For HepG2 cells (Figure 8), at 24 h and 48 h after the treatment, the viability decreased slightly, following free GEM and Fe_3_O_4_@GEM treatments, being around 80%, compared to controls, without a significant dependence on concentration. For the highest equivalent GEM concentration (0.15 mg/mL) the effect produced by the nanoconjugate was statistically more pronounced than the one produced by free GEM. The cytotoxic effect was accelerated up until 72 h after the treatment, the Fe_3_O_4_@GEM being more effective for all the concentration range we tested, the efficiency increasing linearly with the dose. At this time interval, the viability decreased with 40%, in case of Fe_3_O_4_@GEM, compared to free drug, for the highest equivalent concentration (Figure 8C). We may confirm that the effect of Fe_3_O_4_@GEM is more pronounced at longer period of exposure due to the prolonged release of GEM from the nanoparticles surface (Section 2.3.2). Such slower GEM release in the environment was reported for chitosan-GEM coated magnetite nanoparticles [38].

It is known that, for free GEM, the entrapment is done via nucleoside transporters [39], while inorganic nanoparticles can be either entrapped by endocytosis or can directly diffuse into the cells [40]. The cell’s specific interplay of the kinetics of these processes could explain the differences we observed between the cell lines responses.

The third cell line we tested was an interesting particular case. The osteosarcoma MG-63 cell line belongs to the gap junction—positive cell line category. Free GEM proved to be highly cytotoxic for these cells, beginning from low concentrations (Figure 9). The cytotoxic activity of pure GEM was observed to be more prominent than the effect induced by the nanoconjugate. However, the nanosystem’s effects also proved to be highly cytotoxic for the highest equivalent GEM concentration used (0.12 mg/mL), leading to a viability under 40%, after 72 h of treatment. The entrapment of the nucleoside analog appears to be done much quicker, compared to functionalized magnetite, conducting to a higher cell death. A similar high sensitivity to Gemcitabine (viabilities decreased with 50%, compared to controls) was reported for other gap junction−positive cell lines, namely glioblastoma U87 and SKI-1 cell lines, osteosarcoma MNNG/HOS cell line [41]. The increased efficiency of the GEM treatment was proved to be determined by the fact that the diffusion mechanism is done via gap junctions.

Cell viability showed that the transport of the drug may improve the cytotoxic effects in comparison with the one produced by free GEM for the breast cancerous cells and hepatocarcinoma cells, but the magnitude of the effect is cell type dependent (higher in hepatocarcinoma cells than in breast cancerous cells). Moreover, in some particular cells (like gap junction−positive cell lines) the conjugation of GEM with nanoparticles proved to diminish the cytotoxic effect.

A slight resistance of BT 474 and HepG2 cell lines to Gemcitabine treatment can be emphasized from our data (Figure 7 and Figure 8). These results are confirmed by other published studies in which it is shown the same amplitude of GEM cytotoxic effects [42,43]. To overcome this, a bypass of the usual transport pathway for the active substance must be found. For this purpose, the chemotherapeutic substance was attached to the surface of the nanoparticles (or encapsulated); in this case, the as-resulted system was differently perceived by the cells, usually, due to very small dimensions and reactivity, the nanoparticles immediately passing into the cell.

Considering the effects of GEM−conjugated nanoparticles on tumor cell cultures, our results proved that even just a simple direct functionalizing of magnetic nanoparticles with this chemotherapeutic substance produces (1) an increase in the GEM cytotoxicity in BT 474 and HepG2 cells, and (2) the release of GEM is slower at the level of cells. In order to increase GEM half-life in systemic circulation, Viota et al. [15] obtained systems based on magnetite nanoparticles covered with successive layers of poly (acrylic) acid and chitosan. The in vitro effects were assessed for liver, breast and colon cancer cells. Fragmented aspect of the nuclei in treated cells was associated with the fact that Gemcitabine delivery is done at this level. Systems based on albumin/Fe_3_O_4_ nanoparticles/Gemcitabine [16] proved to be more efficient on non-small lung cancer cells by involving magnetic hyperthermia. Wang et al. [17] obtained albumin/Fe_3_O_4_/Gemcitabine nano-spheres conjugated with Cetuximab to be used as multifunctional platforms in targeting pancreatic cancer cells; a similar conclusion was taken regarding the implication of an additional thermo-therapeutic effect.

For further investigations of Fe_3_O_4_@GEM toxic effects, changes in cytoskeleton morphology were searched for [44]. The cytoskeleton is considered to be a “biosensor of cellular well-being” [45], its changes in integrity directly affecting the cells normal functioning, as the cytoskeleton is implied in many cellular functions: (1) cell division (mitosis stage); (2) cytokinesis; (3) cell motility; (4) contraction in muscle cells; (5) endocytosis; (6) intracellular trafficking; (7) protein secretion; (8) normal morphology maintenance; [46].

**Figure 10:**
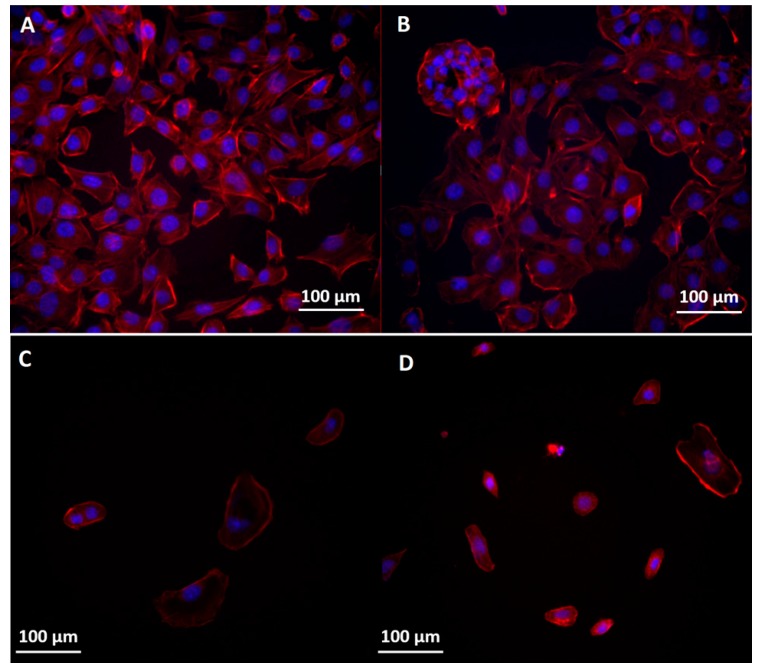
Morphology of the cytoskeleton and nuclei in BT 474 cells: (**A**) control; (**B**) exposed to the highest concentration of Fe_3_O_4_; (**C**) exposed to the highest concentration of Fe_3_O_4_@GEM and (**D**) exposed to the highest concentration of GEM, at 24 h (0.09 mg/mL equivalent concentration of GEM). The cytoskeleton and nuclei were stained with red X-phalloidin and Hoechst.

**Figure 11:**
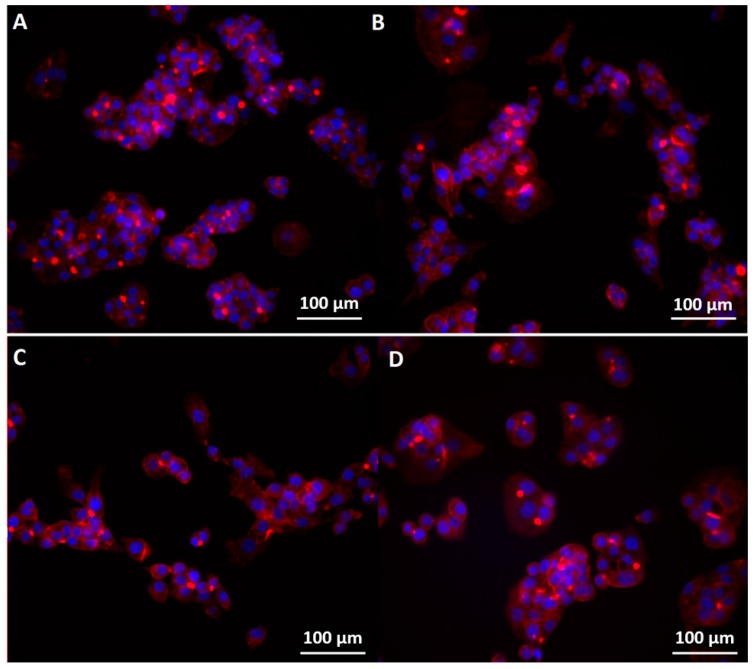
Morphology of the cytoskeleton and nuclei in HepG2 cells: (**A**) control; (**B**) exposed to the highest concentration of Fe_3_O_4_; (**C**) exposed to the highest concentration of Fe_3_O_4_@GEM and (**D**) exposed to the highest concentration of GEM, at 24 h (0.12 mg/mL equivalent concentration of GEM). The cytoskeleton and nuclei were stained with red X-phalloidin and Hoechst.

**Figure 12:**
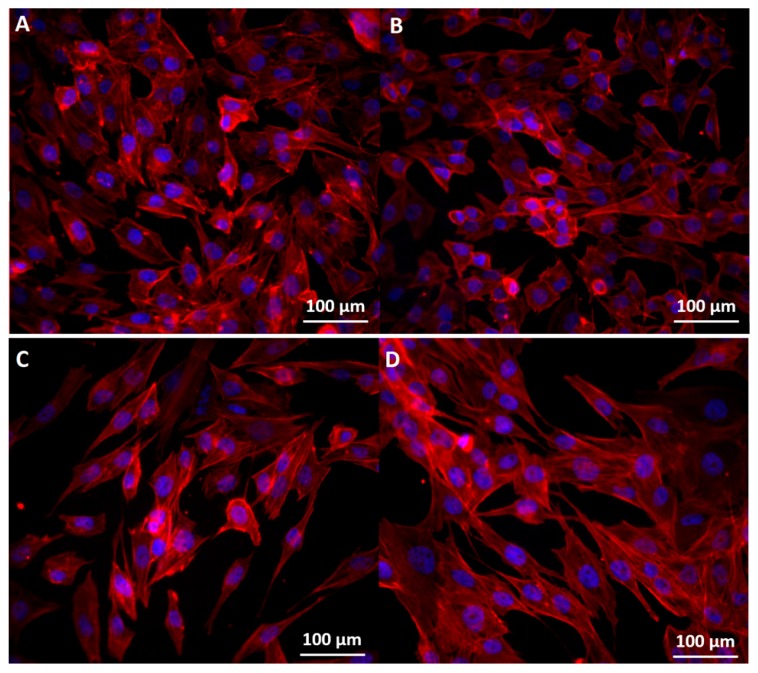
Morphology of the cytoskeleton and nuclei in MG-63 cells: (**A**) control; (**B**) exposed to the highest concentration of Fe_3_O_4_; (**C**) exposed to the highest concentration of Fe_3_O_4_@GEM and (**D**) exposed to the highest concentration of GEM, at 24 h (0.03 mg/mL equivalent concentration of GEM). The cytoskeleton and nuclei were stained with red X-phalloidin and Hoechst.

Immunofluorescence images showed that the nanoconjugate disturbed the regular organization of actin filaments network for all three cell lines (Figure 10, Figure 11 and Figure 12). Orynbayeva et al. [45] appreciated that, in primary endothelial cells, the magnetic nanoparticles determine a local displacement in the continuous actin filaments and microtubule network rather than breaking them. These displacement in the cells cytoskeleton were also observed after the treatment of endothelial cells with ZnO nanoparticles [47], being called “actin clumps”.

The morphology of BT 474 cells was severely affected by the treatment with Fe_3_O_4_@GEM and GEM alone; the cytoplasm lost its integrity and the nuclei appeared as inconsistent (the Hoechst stain fainted) or appeared scattered (Figure 10). The cells treated with Fe_3_O_4_ nanoparticles were more round compared to control samples, with an increased actin concentration similar to “actin clumps”, at the extremities of the cells’ cytoplasm.

For HepG2 cells (Figure 11), the organization in bunch-like clusters which normally appears in control cells was almost lost after the treatment with the functionalized magnetite nanoparticles and the chemotherapeutic substance alone; in normal conditions, the cytoplasm of HepG2 cells has a consistency much alike jellyfishes; after interacting with both functionalized and unfunctionalized nanoparticles, the morphology of the cells was more elongated, however there were differences in the cellular detachment (which was more pronounced after the functionalizing of the nanoparticles); the nuclei were not affected.

In case of MG 63 cells (Figure 12), exposed to bare Fe_3_O_4_ nanoparticles, the detachment of the cells was poorly noticeable, but the cells were round and smaller than in control samples. For cells treated with functionalized nanoparticles or GEM alone, besides the cells detachment, a higher concentration of actin was evidenced in certain areas around the nuclei of the cells or around the extremities and the nuclei were larger in diameters.

**Figure 13:**
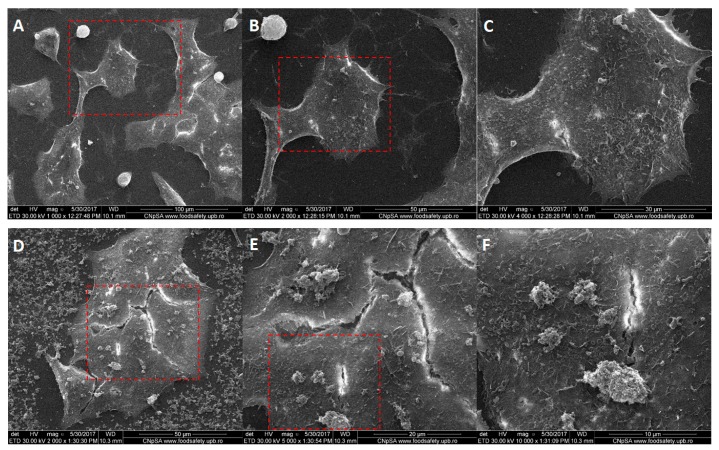
Morphology of BT 474 cells: (**A**–**C**) control, at different magnifications (1000×, 2000×, 4000×); (**D**–**F**) exposed to the highest concentration of Fe_3_O_4_@GEM, at different magnifications (2000×, 5000×, 10,000×), after 24 h of exposure; red squares represent the area of magnification.

**Figure 14:**
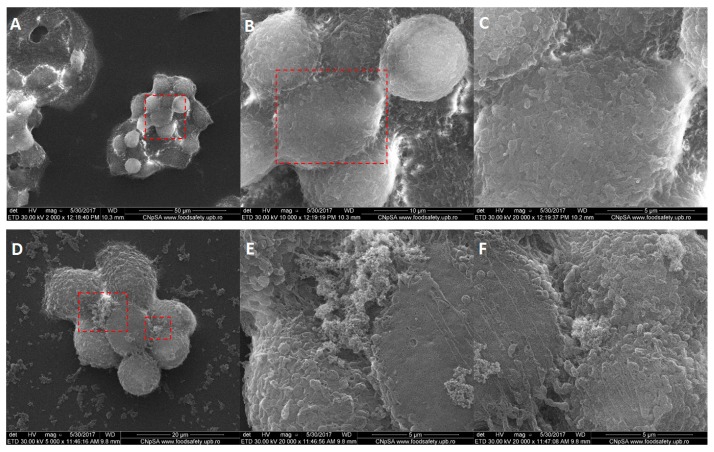
Morphology of Hep G2 cells: (**A**–**C**) control, at different magnifications (2000×, 10,000×, 20,000×); (**D**–**F**) exposed to the highest concentration of Fe_3_O_4_@GEM, at different magnifications (5000× and 20,000×), after 24 h of exposure; red squares represent the area of magnification.

**Figure 15:**
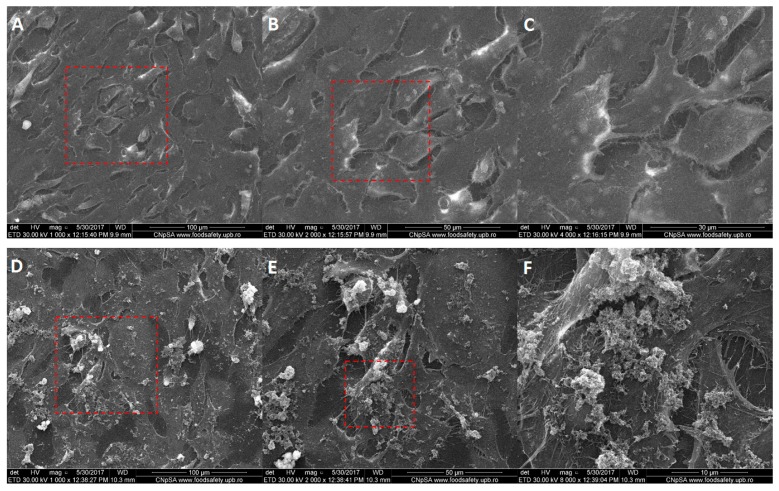
Morphology of MG-63 cells: (**A**–**C**) control, at different magnifications (1000×, 2000×, 4000×); (**D**–**F**) exposed to the highest concentration of Fe_3_O_4_@GEM, at different magnifications (1000×, 2000×, 8000×), after 24 h of exposure; red squares represent the area of magnification.

Scanning electron microscopy imaging was employed to complete the results from fluorescence microscopy regarding the membrane morphology of the tumor cells after their interaction with Fe_3_O_4_@GEM nanoparticles, results which were in concordance with the previous ones. The morphology of BT 474 cells (Figure 13) is not affected after being exposed for 24 h to functionalized nanoparticles, the presence of Fe_3_O_4_@GEM being evidenced at the exterior cell, as agglomerates with sub-micrometric dimension, directly interacting with the cellular membrane (Figure 13E,F). The bright areas at the extremities of the cells are given by the reflection of electrons and might be due to the increased actin concentration observed in fluorescence microscopy (Figure 10). In case of Hep G2 (Figure 14) and MG-63 (Figure 15), the morphology of the cells is not affected by their interaction with the nanostructured systems, the presence of the nanoaparticles agglomerates being clearly evidenced in case of osteosarcoma cells (Figure 15F).

**Figure 16:**
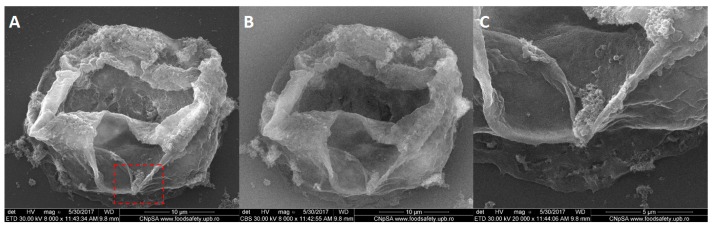
Morphology of a Hep G2 cell which occurred in death: (**A**) image resulted using secondary electrons signal (magnification 8000×); red square represents the area of magnification; (**B**) image resulted using back scattered electrons (magnification 8000×); (**C**) magnification (20,000×) of A; detail of Fe_3_O_4_@GEM nanoparticles inside the fragmented cell.

Figure 16 shows the morphology of a necrotic Hep G2 cell, which suffered cell death after being exposed to Fe_3_O_4_@GEM (in the highest concentration, during 24 h). The breakage of the cell membrane revealed the presence of the nanoparticles aggregates inside the folds of the membrane.

**Figure 17:**
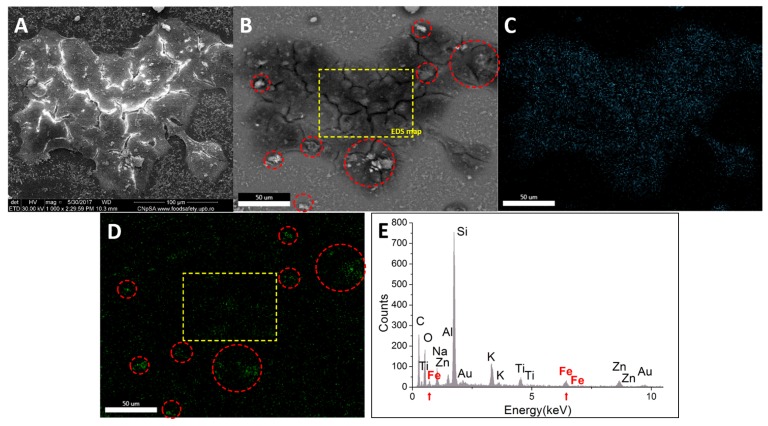
Qualitative and quantitative elemental characterization of BT 474 cells exposed to the highest concentration of Fe_3_O_4_@GEM, after 24 h of treatment: (**A**) morphology image resulted using secondary electrons signal (magnification 1000×); (**B**) morphology image resulted using back scattered electrons signal (magnification 1000×); (**C**) carbon elemental mapping on B; (**D**) iron elemental mapping on B; (**E**) EDX spectrum on B (yellow square); red circles mark the extracellular agglomerates of nanoparticles; yellow square marks an area free of extracellular nanoparticles, which was subjected to quantitative elemental analysis; red arrows mark the presence of Fe in the EDX subjected area.

Energy dispersive X-ray spectroscopy was employed in order to evaluate the localization of the nanostructured systems after being in contact with tumor cells for 24 h. In case of BT 474 (Figure 17), the morphology image resulted using secondary electron signal (Figure 17B) evidences the presence of nanoparticle aggregates at the exterior of the cells (marked with red circles); this mode of analysis showcases the elements with higher atomic number as lighter (like Fe), while the elements with low atomic number (like C) are represented in darker shades. The yellow square (Figure 17B) delimitates an area which was free of bright elements, thus no extracellular iron oxide nanoparticle agglomerates (as compared with the morphology image resulted using secondary electrons signal—Figure 17A). This area was subjected to EDX mapping, Figure 17C showing the carbon element, which is present in cells, respectively Figure 17D showing the iron element from the nanoparticles. Similarly to Figure 17B, in Figure 17D were evidenced the extracellular aggregates of Fe_3_O_4_@GEM using red squares (green dots with higher intensity).

The yellow square emphasizes the presence of Fe agglomerates which correspond to the area free of magnetite nanoparticles in Figure 17B; these Fe agglomerates are covered with an organic layer (as seen in Figure 17B). Also, the area marked with the yellow square was subjected to quantitative EDX, the elemental atomic composition showing a concentration of 0.33 at·% of Fe, with an error of 18.47%.

**Figure 18:**
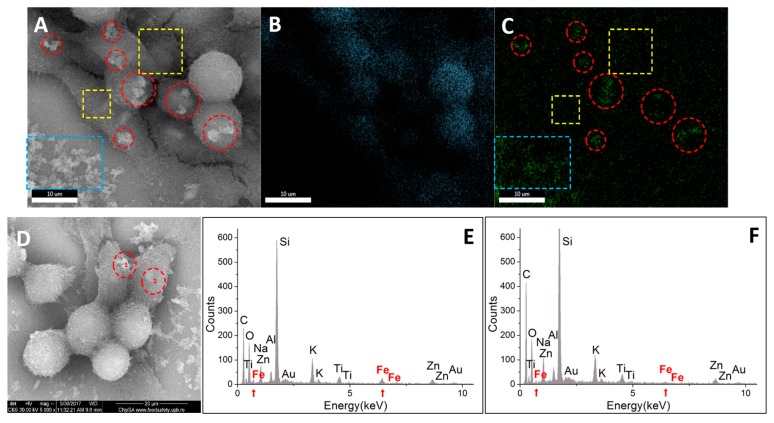
Qualitative and quantitative elemental characterization of Hep G2 cells exposed to the highest concentration of Fe_3_O_4_@GEM, after 24 h of treatment: (**A**) morphology image resulted using secondary electrons signal (magnification 10,000×); (**B**) carbon elemental mapping on A; (**C**) iron elemental mapping on A; (**D**) morphology image resulted using secondary electrons signal (magnification 5000×); 1—area with aggregates of nanoparticles, situated in the exterior of the cell membrane; 2—area free of elements with high atomic number; (**E**) EDX spectrum on D (area 1); (**F**) EDX spectrum on D (area 2); red circles mark the extracellular agglomerates of nanoparticles; yellow square marks an area free of extracellular nanoparticles; blue square marks an area of nanoparticles attached to the glass substrate; red arrows mark the presence of Fe in the EDX subjected area.

Same behavior as in case of BT 474 cells can be emphasized for Hep G2 cells (Figure 18). Here, the EDX quantitative evaluation was done for two areas which are clearly distinctive by means of the localization of the nanoparticles: area 1 shows bright aggregates of nanoparticles, situated in the exterior of the cell membrane, while area 2 shows a darker area free of heavier elements (Figure 18D). The results for quantitative elemental composition showed a concentration of 0.25 at% Fe, error 23.09%, in area 1, respectively of 0,06 at% Fe, error 62,01% in area 2.

**Figure 19:**
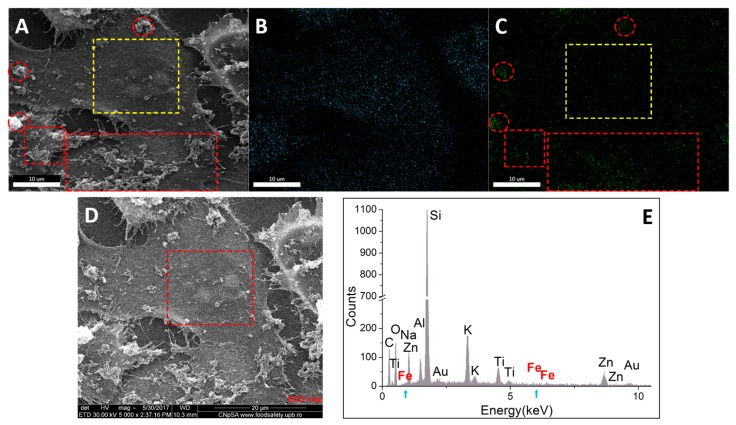
Qualitative and quantitative elemental characterization of MG-63 cells exposed to the highest concentration of Fe_3_O_4_@GEM, after 24 h of treatment: (**A**) morphology image resulted using secondary electrons signal (magnification 5000×); red circles and red squares mark the extracellular agglomerates of nanoparticles; yellow square an area free of extracellular nanoparticles; (**B**) carbon elemental mapping on A; (**C**) iron elemental mapping on A; (**D**) morphology image resulted using secondary electrons signal (magnification 5000×); red square marks the area, which was subjected to quantitative elemental analysis; (**E**) EDX spectrum on D (red square); blue arrows mark the absence of Fe in the EDX subjected area.

In case of MG-63 cells (Figure 19), is evidenced the disparity between the areas with extracellular aggregates of Fe_3_O_4_@GEM (delimited with red dots) and an area with no extracellular nanoparticle aggregates (yellow square). The EDX mapping and EDX quantitative spectrums showed no presence of Fe in the selected area; the faded green dots in Figure 19C are due to the background noise. These results suggest the fact that the nanoparticles may not be internalized in MG-63 cells, being in concordance with the viability measurements, which showed low cytotoxic effects (Figure 9).

**Figure 20:**
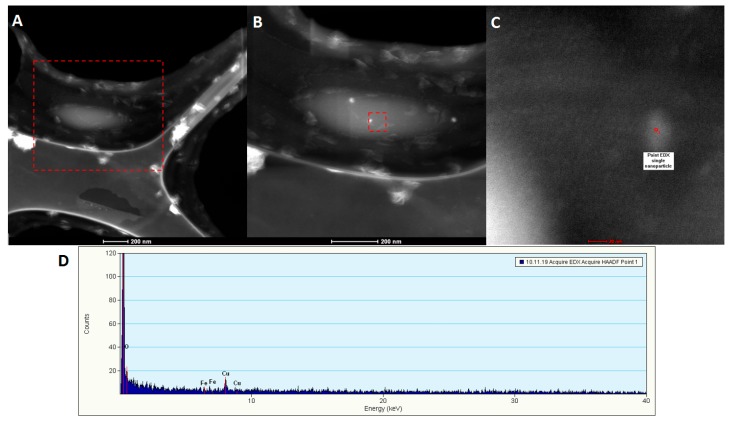
Qualitative and quantitative elemental characterization of BT 474 cells exposed to the highest concentration of Fe_3_O_4_@GEM nanoparticles, after 24 h of treatment: (**A**) STEM image for BT 474 cell morphology, red square represents the area of magnification; (**B**) detail of A with emphasize on nucleus of the cell; (**C**) detail of single Fe_3_O_4_ functionalized nanoparticle, internalized into the cell; (**D**) EDX spectrum of single Fe_3_O_4_ nanoparticle internalized in the cell.

Furthermore, we employed scanning transmission electron microscopy analysis (STEM), in order to prove the internalizing of the nanoparticles inside the tumor cells. Here are given the results for BT 474 cells, as considering the fact that they showed the best results for previous studies. Figure 20A shows a breast cancer cell attached to the carbon grid used for transmission electron analysis; the nucleus of the cell is represented with brighter shades, due to the fact that it is a more condensed area than the cytoplasm (darker area).

In Figure 20B the light spots are artefacts (due to electron reflections), however in Figure 20C is evidenced the presence of a single nanoparticles which was activated in the electron fascicle. In order to prove the composition of this area, single nanoparticle EDX was employed, showing the presence of Fe and O elements. The quantitative measurements showed a concentration of 0.05 at·% Fe, with an error of 0.03%.

The hemolytic test is a recognized sensitive test that plays an important role in evaluation of biosafety. This test is used to measure the biocompatibility of a biomaterial by direct contact with blood. Our study shows that the positive control induced a massive hemolysis (~100%), while our samples of nanoconjugate induced erythrocytes lysis at a level similar with the negative control and DPBS. The hemolysis level is less than 5% as recommended by ASTM E2524-08 standard. These results demonstrated the good biocompatibility of our material. The hemocompatibility of magnetite nanoparticles nanoconjugates based on magnetite nanoparticles was proved also in other studies using either whole blood or isolated erythrocytes [36,48,49].

**Figure 21:**
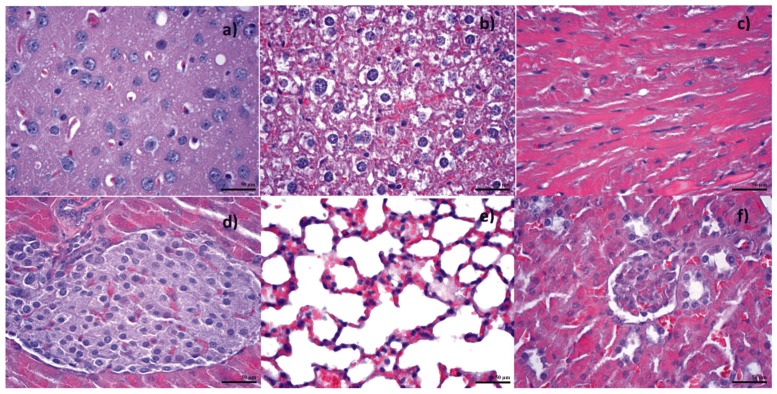
Transversal section through (**a**) brain; (**b**) liver; (**c**) myocardium; (**d**) pancreas; (**e**) lungs; (**f**) kidneys; from mice injected with Fe_3_O_4_@GEM; samples collected at 7 days after the treatment; normal morphology; Hematoxilin-Eosin staining (400× magnification).

**Figure 22:**
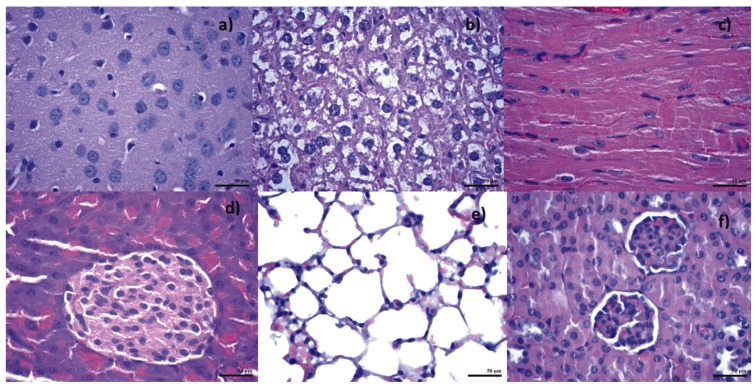
Transversal section through (**a**) brain; (**b**) liver; (**c**) myocardium; (**d**) pancreas; (**e**) lungs; (**f**) kidneys; from mice injected with Fe_3_O_4_@GEM; samples collected at 14 days after the treatment; normal morphology; Hematoxilin-Eosin staining (400× magnification).

**Figure 23:**
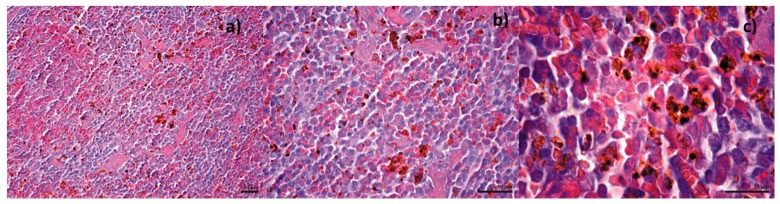
Transversal section through spleen from mice injected with Fe_3_O_4_@GEM; samples collected at 7 days after the treatment; normal morphology; Hematoxilin-Eosin staining, magnification: (**a**) 200×; (**b**) 400×; (**c**) 1000×.

**Figure 24:**
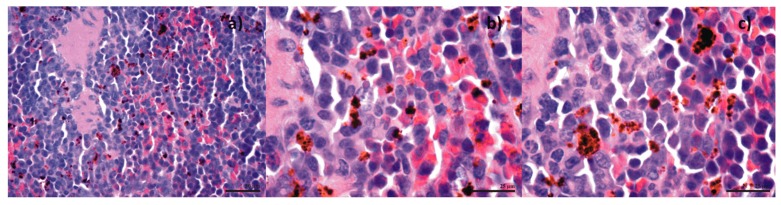
Transversal section through spleen from mice injected with Fe_3_O_4_@GEM; samples collected at 14 days after the treatment; normal morphology; Hematoxilin-Eosin staining, magnification: (**a**) 400×; (**b**,**c**) 1000×.

Following in vitro bioevaluation of this novel nanoconjugate, an in vivo study was performed, in order to see the biodistribution of the nanoparticles in the vital organs. The results from the biodistribution study were obtained on albino mice, which were intraperitoneally injected with Fe_3_O_4_@GEM, showing no evidence of nanoparticle accumulation in organs like the brain, liver, heart (myocardium), lungs or pancreas for subjects evaluated at both 7 and 14 days from the treatment (Figure 21 and Figure 22). Instead, the presence of nanoparticles was evidenced at spleen level, as well defined aggregates, for both time intervals. At 7 days after the treatment (Figure 23), the nanoparticles were evidenced in the red pulp, being absent from the white pulp, but it appeared to be hypertrophied; this phenomenon is determined by the fact that the presence of nanoparticles into the systemic circulation stimulates the macrophages. At red pulp level, the nanoparticles were evidenced inside the macrophage cells, present into Billroth cordons, but also inside the sinusoid capillary. Fe_3_O_4_ nanoparticles appear aggregated, like dark brown spherical granular structures, with variable dimensions (diameters up to 13 µm). At 14 days after the treatment (Figure 24), a higher density of magnetite aggregates was evidenced into the red pulp.

Intraperitoneal delivery is preferred for local cancer therapy due to the anatomical peculiarities of the peritoneum. Healthy tissues outside the peritoneal cavity are protected from the side effects following the administration of nanoparticles functionalized with antitumor drugs, mainly for ovarian and gastric cancer [50,51,52]. Also, intraperitoneal injection improves the uptake of NPs-labeled high-density lipoprotein to atherosclerotic plaques compared with intravenous injection in ApoE knockout mice [53].

Two time periods (7 and 14 days) were chosen to better emphasize the bioaccumulation of Fe_3_O_4_@GEM NPs and the protection of healthy tissues outside the peritoneal cavity. Following NPs administration, there is no evidence of NPs accumulations in organs like brain, liver, myocardium, lung or pancreas. This biodistribution study is relevant for the fact that one must know where the nanoparticles tend to deposit in the absence of an active targeting, like magnetic guidance or specific functionalizing. Also, it is important to know where the nanoparticles tend to go after they carry out the action for which they were designed and their interaction with those living structures.

## 3. Materials and Methods

### 3.1. Nanoparticle Synthesis

In order to obtain the (un) functionalized magnetite nanoparticles, the following substances were purchased from Sigma Aldrich ChemieGmbh (Saint Louis, MO, USA): ferrous sulfate 7-hydrate (FeSO_4_·7H_2_O), ferric chloride (FeCl_3_), 25% ammonia solution (NH_3_) and Gemcitabine (GEM). All chemicals were of analytical purity and used with no further purification. For the synthesis of the (un) functionalized magnetite nanoparticles, a derived chemical co-precipitation method was employed: FeSO_4_·7H_2_O (1.2 g) and FeCl_3_ (2 g) were dissolved in ultrapure water (400 mL) to form the precursor solution and 25% NH_3_ solution (5 mL) was mixed with ultrapure water (200 mL) to obtain the precipitation medium; in order to obtain functionalized nanoparticles, 100 mg Gemcitabine were also dissolved into the ammonia solution; the precursor solution was added dropwise into the precipitation medium, under magnetic stirring. The resulted colloids (Gemcitabine functionalized, respectively unfunctionalized magnetite nanoparticles-Fe_3_O_4_@GEM and Fe_3_O_4_) were magnetically separated and washed several times with ultrapure water.

### 3.2. Structural Characterization

#### 3.2.1. X-ray Diffraction

In order to evaluate the samples crystallinity using X-ray Diffraction Analysis (XRD), we used a XRD 6000 difractometer (Shimadzu, Kyoto, Japan). The scanning was done at room temperature, using a Cu Kα = 1.056 Å (15 mA and 30 kV) and a Bragg angle of 2θ = 10–80° (degree).

#### 3.2.2. Thermogravimetric Analysis

The thermogravimetric analysis was done using a Shimadzu DTG-TA-50H instrument (Shimadzu, Kyoto, Japan). The samples were placed into platinum crucibles and screened to 200 mesh prior to analysis: after that, the samples were heated with 10 K·min^−1^, from room temperature to 800 °C, under flow of 20 mL/min, in dried synthetic air (80% N_2_ and 20% O_2_).

#### 3.2.3. Scanning Electron Microscopy and Energy Dispersive X-ray Spectroscopy

The scanning electron microscopy analysis was assessed using a FEI electronic microscope (FEI Company, Hillsboro, OR, USA), equipped with EDX, with a beam of secondary electrons having energies up to 30 keV. The samples were prepared by placing onto a carbon strip attached to a metallic stand.

#### 3.2.4. Transmission Electron Microscopy, High Resolution Transmission Electron Microscopy and Selected Area Electron Diffraction

The transmission electron microscopy was done using a Tecnai™ G2 F30 S-TWIN HR-TEM (FEI Company, Hillsboro, OR, USA) equipment with selected area electron diffraction (SAED). The preparation step of the samples consisted in dispersing the powders in ethanol, followed by sonication during 15 min and successive dilutions of the resulted suspension, in order to result low concentrations suitable for the analysis. The following step consisted in placing the suspension onto a holey carbon-copper grid and drying. The equipment was set to transmission mode at 300 kV, with 2 Å point resolution and 1 Å line resolution.

### 3.3. Interaction with Biologic Media

#### 3.3.1. Dynamic Light Scattering and Zeta Potential Measurements

The hydrodynamic diameter and surface charge (zeta potential) of GEM functionalized Fe_3_O_4_ nanoparticles dispersed in both types of biological cell culture media—DMEM and MEM—were characterized using a Delsa^TM^ Nano C instrument (Beckman Coulter, Brea, CA, USA). The measurements were done for freshly prepared nanoparticles and dilutions, prepared by ultrasound dispersion (same as in Section 3.4). The analysis of the particles, their size distribution and stability in suspension solutions was done in accordance with the existing international documentary standards (ASTM 2009), as well as ISO 13321 (ISO 1996) and more recently ISO 22412 (ISO 2008b), where the measured particle size distribution is assumed unimodal, and it fits a single exponentially decaying function (corresponding with one particle size) to the measured autocorrelation function. Based on the translational diffusion coefficient information, the method gives information about a mean diameter and an estimative width of the distribution (PDI, or polydispersity index), a parameter that is only meaningful where the sample’s size distribution is unimodal. All measurements were recorded in triplicate at 25 °C and for data analysis, and the DelsaNano 3.73 software (Beckman Coulter, Brea, CA, USA) was used.

#### 3.3.2. Release Kinetics

In order to evaluate the kinetics of Gemcitabine release from Fe_3_O_4_@GEM conjugates, a suspension of 1% nanoparticles in water was dried by magnetic separation and then suspended in complete DMEM culture media, in order to obtain the same concentration of nanoparticles. The samples were incubated for different time intervals in standard conditions (37 ± 2 °C, 5 ± 1% CO_2_, more than 90% humidity); the release of GEM in culture media was emphasized in the interval of 300–200 nm, as the characteristic peak for gemcitabine (270 nm) was shifted towards 250 nm. Two standards of GEM in complete DMEM were prepared (having concentrations of 0.01 respectively 0.05 mg/mL). The measurements were done using a Cary 100 Bio UV-VIS Spectrophotometer (Varian Medical Systems, Pao Alto, CA, USA) and the spectra were recorded using the Cary WinUV Scan software (Agilent Technologies, Santa Clara, CA, USA).

### 3.4. Biological Evaluation

#### 3.4.1. In Vitro Studies

The in vitro cytotoxicity of the designed nanoparticles was assessed for several human cancerous cell lines: BT 474 human breast ductal carcinoma, HepG2 human hepatocellular carcinoma and MG 63 human osteosarcoma cells lines, which were purchased from ATCC^®^ (Mansas, VA, USA). BT 474 and HepG2 cells were cultured in Dulbecco’s modified Eagle’s medium (DMEM) (Biochrom, Merck Milipore, Billerica, MA, USA), supplemented with 10% fetal bovine serum (Biochrom) and 1% antibiotics (penicillin and streptomycin) (Biochrom), while the MG 63 cells were cultured in MEM Earle’s (MEM) (Biochrom), supplemented with 10% fetal bovine serum (Biochrom), 1% L-glutamine (Biochrom), 1% non-essential amino-acids (Sigma Aldrich) and 1% antibiotics (penicillin and streptomycin) (Biochrom).

##### Cytotoxicity Assay

The quantitative evaluation of the effect was done using the tetrazolium-salt (MTS) based viability assay, CellTiter 96^®^ AQ_ueous_ One Solution Cell Proliferation Assay (Promega, Madison, WS, USA), according to the producer’s specifications. For this, 10,000 cells/well were seeded in 96-well plates and cultured for 24 h in standard conditions (37 ± 2 °C, 5 ± 1% CO_2_, more than 90% humidity). Meanwhile, solutions of Fe_3_O_4_, Fe_3_O_4_@GEM and Gemcitabine alone in equivalent concentrations were prepared by ultrasound dispersion/dilution in complete culture medium (binary dilutions of 0.15 mg/mL Gemcitabine; the equivalent concentrations of Fe_3_O_4_@GEM/Fe_3_O_4_ controls were calculated from the thermogravimetric analysis data); 100 µL of the prepared solutions were added in each of the sample wells, while in control wells (untreated cells) complete culture medium was added; blank samples were also prepared (wells with no cells) in order to eliminate possible interferences. The viability measurements were done at 24, 48, respectively 72 h after the treatment. For this, the nanoparticles were gently removed from the wells and 20 µL of MTS solution was added over 100 µL culture medium (DMEM/MEM complete, supplemented with 5% fetal bovine serum) in each well. The plates were incubated for 3 h in standard conditions. The absorbance was read at 490 nm using a Sunrise microplate reader (Tecan, Männedorf, Switzerland) directly into the plate or in supernatant.

##### Cell Morphology and Cytoskeleton Evaluation

For the qualitative evaluation of the cytotoxic effects, the treated cells morphology and cytoskeleton integrity were evaluated using Texas Red^®^-X Phalloidin (ThermoFisher Scientific, Waltham, MA, USA) fluorescence staining. For this purpose, 10,000 cells were seeded on each 10 mm diameter glass slides placed in 24-well plates and cultured for 24 h in standard conditions. The treatment was done similarly as to the cytotoxicity assay; 300 µL of the prepared solutions were added in each of the sample wells, while in control wells (untreated cells) complete culture medium was added. The samples were evaluated at 24, 48 and 72 h after the treatment. To prepare the slides for fluorescence microscopy, the nanoparticles were removed from the wells and the cells were gently washed with phosphate buffer saline (PBS) three times; then, 3.7% paraformaldehyde solution was added for fixing, for 5 min. Next, the cells were permeabilized with 1% Triton X for 10 min and colored with Texas Red-Phalloidin for 40 min and Hoechst for 10 min (at dark). The cells were gently washed with PBS after each step. The visualization of the as-prepared samples was done using an LX71 fluorescence microscope (Olympus, Shinjuku, Tokyo, Japan) and the image recording was done using ixon+ image recorder (Andor Technology, Belfast, UK).

##### Scanning Electron Microscopy and Energy Dispersive X-ray Spectroscopy

Scanning electron microscopy and energy dispersive X-ray spectroscopy were employed to assess the morphology and elemental composition of tumor cells, previously exposed to Fe_3_O_4_@GEM. The seeding and nanoparticle treatment were done as for Cell Morphology and Cytoskeleton Evaluation analysis. At 24 h after the treatment, the samples were washed with PBS for 3 times and fixed with glutaraldehyde 2.5% for 1 h. After washing, the cells were dehydrated with ethanol (Sigma Aldrich) solutions with increasing concentration (70, 90, respectively 100%), for 15 min, by repeating two times each step. After this, different ethanol:hexamethyldisilazane HMDS (Sigma Aldrich) solutions were used to further dehydrate the cell samples (concentrations of ethanol:HMDS of 50:50, 25:75, respectively 0:100%); the treatment was applied for 3 min, by repeating two times each step. The samples were covered with a thin layer of Au and subjected to analysis using a FEI electronic microscope, equipped with EDX, with a beam of secondary electrons having energies up to 30 keV.

##### Scanning Transmission Electron Microscopy and Energy Dispersive X-ray Spectroscopy

Scanning transmission electron microscopy and energy dispersive X-ray spectroscopy were used in order to prove the internalizing of Fe_3_O_4_@GEM in tumor cells. For this, 100,000 BT 474 cells were cultured for 24 h in 24-well plates and, after this period of time, the culture medium was replaced with fresh medium with nanoparticles in the highest concentration of nanoparticles, which was used in the study. The samples were incubated for another 24 h and, then the cells were washed 3 times with PBS, in order to remove the excess of nanoparticles, then detached using trypsin and suspended in fresh complete medium. The samples were washed with PBS by centrifugation at 400 rpm, during 5 min and fixed with glutaraldehyde 2.5% for 1 h. After washing, the cells were dehydrated with ethanol (Sigma Aldrich) solutions with increasing concentration (70, 90, respectively 100%), for 15 min, by repeating two times each step. The final dilution was done in ethanol 100% and the following step consisted in placing the suspension onto a holey carbon-copper grid and drying. The transmission electron microscopy was done using a Tecnai™ G2 F30 S-TWIN HR-TEM (FEI Company) equipment with selected area electron diffraction (SAED). The equipment was set to scanning transmission mode at an accelerating voltage of 300 keV, with an energy resolution of 134 eV and a beam current of 1 nA.

##### Hemolysis Assay

The hemolytic potential of GEM functionalized magnetite nanoparticles was evaluated by using a standard test described in detail in the ASTM standard E2524-08 (Standard test method for analysis of Hemolytic properties of nanoparticles). A mixture of freshly heparinized whole blood from 3 human healthy donors was used. Plasma free hemoglobin (PFH) and total blood hemoglobin (TBH) was determined using Drabkin’s reagent. The blood was diluted to adjust TBH to 10 ± 1 mg/mL with Ca/Mg free Dulbecco’s PBS (DPBS).

The nanoparticles samples were prepared using a nanoparticles stock solution, by washing the nanoparticles from the original solvent (distilled water) by magnetic separation, followed by resuspension in DPBS. The nanoparticle solution was well mixed and sonicated for 15 min. Serial 1:5 dilutions were made to get a final concentration ranged from 2.5 mg/mL to 0.02 mg/mL.

This method is based on quantitative determination of hemoglobin released into PFH as a percentage of the TBH concentration when blood is exposed in our case to GEM functionalized magnetite nanoparticles.

The hemolytic potential was determined using as a cyan methemoglobin reagent Drabkin’s solution. Positive control (triton X-100 1% in distilled water), negative control (PEG8000) and blank (DPBS) were tested similar to the samples. The blood-samples solutions were incubated for 3 h at 37 °C, mixing the tubes every 30 min. The tubes were centrifuged and then the supernatants were mixed with Drabkin’s reagent. The samples mean absorbance at 540 nm that revealed hemoglobin concentration were used to calculate percent hemolysis (% hemolysis = PFH_sample_/TBH_diluted_ × 100%). Sample absorbance was corrected for background interference (i.e., particles in DPBS without blood). All the acceptance criteria described in the ASTM standard were followed.

#### 3.4.2. In Vivo Biodistribution Studies

The animal study was approved by the Institutional Ethics Committee of University of Medicine and Pharmacy of Craiova (Approval No. 73/29.05.2014) and was conducted according to national and EU regulations in the field. The experimental protocol was applied according to the European Council Directive No. 86/609/24 November 1986, the European Convention on the Protection of Vertebrate Animals (2005) and the Romanian Government Ordinance No. 37/2 February 2002. We used male C57BL/6 mice, 8–10-week-old, weighing 18–22 g. The in vivo biodistribution of the Fe_3_O_4_ and Fe_3_O_4_@GEM nanoparticles was done for 12 albino mice (each group of four mice), housed in the Animal Care Unit of University of Medicine and Pharmacy of Craiova and were maintained throughout experiments at 22 ± 20 °C, 55 ± 10% humidity and a 12 h light dark cycle. Mice were fed with standard rodent diets, and received food and water *ad libitum*. Each subject was intraperitoneally injected with 1 mg/mL suspension of nanoparticles in PBS, respectively with physiologic serum, for controls. The subjects were divided in two categories, half of them being sacrificed at 7 days after the treatment and the other half at 14 days after the treatment. The animals were anesthetized using commercial euthanasia solution—Euthasol^®^ (390 mg pentobarbital sodium and 50 mg phenytoin sodium per mL; Le Vet. Pharma BV, Oudewater, The Netherlands) 0.22 mL/kg b.w., i.p. (~86 mg/kg b.w. pentobarbital) and the main organs were harvested for histological examination (brain, liver, heart, pancreas, lungs, kidneys, spleen). The organs were washed in PBS, preserved in formaldehyde for several days, embedded in paraffin blocks and sectioned using a microtome. The samples underwent Hematoxilin-Eosin staining and were analyzed using Nikon Eclipse 55*i* (Nikon Instruments, Burgerweeshuispad, Amsterdam, The Netherlands) optical microscope.

#### 3.4.3. Statistics

Values were presented as means ± standard error of the mean. Data were analyzed statistically using a two-ways ANOVA test.

## 4. Conclusions

Besides their specific magnetic properties [54], magnetite (Fe_3_O_4_) nanoparticles are friendly to biological tissues and fluids, the biocompatibility [34,35,36,55] making them promising candidates for numerous biomedical applications, like: (1) drug delivery systems [56,57,58,59]; (2) hyperthermia cancer treatment [60,61,62,63,64]; (3) contrasting agent in magnetic resonance imaging [11,27,65,66]; (4) inhibition of biofilm development [67,68,69,70,71,72,73].

In this article we report the successful preparation and characterization of Fe_3_O_4_ nanoparticles, directly functionalized with the chemotherapeutic substance Gemcitabine, using no additional linker between the two components. As far as we know, there are no similar systems reported in the literature, involving this anti-tumor substance, the only existing data referring to the obtaining of polymeric microparticles separately encapsulating the Gemcitabine and magnetite. These particles have large dimensions and are susceptible to be rapidly detected by the immune system and eliminated from the body before reaching the tumor site. Our obtained Fe_3_O_4_ nanoparticles have physical dimensions under 20 nm, high biocompatibility with human blood and high stability in standardized culture media, for hydrodynamic diameters below 300 nm.

The in vitro biological evaluation of these nanoparticles showed promising results regarding their cytotoxicity against human cancer cells, while the in vivo biodistribution test showed their biocompatibility with normal living tissues. However, in order to have more precision in the administration and reaching the tumor tissue, an active targeting agent may be used. To sum up, the novelty of this system and cytotoxic effects against cancer cells, make from our Gemcitabine−conjugated magnetite nanoparticles promising candidates in the improvement of anti-cancer therapeutic approaches.
